# Extracellular nicotinamide phosphoribosyltransferase (NAMPT/visfatin) inhibits insulin-like growth factor-1 signaling and proteoglycan synthesis in human articular chondrocytes

**DOI:** 10.1186/ar3705

**Published:** 2012-01-30

**Authors:** Raghunatha R Yammani, Richard F Loeser

**Affiliations:** 1 Department of Internal Medicine, Section of Molecular Medicine, Wake Forest School of Medicine, Medical Center Blvd, Winston-Salem, NC 27157, USA

## Abstract

**Introduction:**

Obesity is one of the major risk factors for the development of osteoarthritis (OA). Although the mechanical factors appear to be critical, recent studies have suggested a role for adipokines in cartilage degradation. Chondrocytes from osteoarthritic cartilage respond poorly to insulin-like growth factor-1 (IGF-1) and the molecular mechanism(s) involved is not clearly understood. The purpose of the present study was to determine the role of extracellular nicotinamide phosphoribosyltransferase (eNAMPT/visfatin), a newly described adipokine, in regulating IGF-1 function in chondrocytes.

**Methods:**

Human articular chondrocytes isolated from normal ankle cartilage were pretreated with eNAMPT (0.1 to 5.0 μg/ml) overnight followed by stimulation with IGF-1 (50 ng/ml) for 24 hours, and proteoglycan synthesis was measured by [^35^S]sulfate incorporation. Chondrocytes were pretreated with eNAMPT overnight followed by IGF-1 for 10 minutes, and the cell lysates were immunoblotted for various signaling proteins that are activated by IGF-1 using phosphospecific antibodies. In addition, chondrocytes were pretreated with mitogen-activated protein kinase kinase inhibitor (U0126) prior to stimulation with eNAMPT and IGF-1.

**Results:**

Pretreatment of chondrocytes with eNAMPT inhibited IGF-1-stimulated proteoglycan synthesis in a dose-dependent manner. Treatment of chondrocytes with eNAMPT inhibited IGF-1-induced phosphorylation of signaling molecules, including insulin receptor substrate-1 and AKT. Interestingly, pretreatment of chondrocytes with eNAMPT did not inhibit IGF-1-mediated phosphorylation of the IGF-1 receptor; however, it stimulated a sustained phosphorylation of the extracellular signal-regulated kinase (ERK)/mitogen activated protein kinase (MAPK) signaling pathway. Inhibition of the ERK/MAPK signaling pathway restored IGF-1-mediated insulin receptor substrate-1 and AKT phosphorylation.

**Conclusions:**

Our study demonstrates that eNAMPT/visfatin inhibits IGF-1 function in articular chondrocytes by activating the ERK/MAPK pathway independent of the IGF-1 receptor. Since eNAMPT levels are elevated in the synovial fluid of OA patients, the signaling pathway activated by eNAMPT could contribute to IGF-1 resistance in OA.

## Introduction

Obesity is a major risk factor for the development of osteoarthritis (OA) [1,2]. Emerging data have shown that metabolic factors associated with obesity, including adipokines, play an important role in the progression of OA, prompting some to classify OA as a metabolic disease. Several adipokines, including leptin, resistin, and adiponectin, have been found in synovial fluid from patients with OA, and are thought to have local effects on joint tissues [3]. Leptin induces IL-1β, matrix metalloproteinase-9 and matrix metalloproteinase-13 expression in chondrocytes [4]. Likewise, adiponectin induces expression of nitric oxide synthase-2, IL-6, monocyte chemoattractant protein-1 and matrix metalloproteinases [5]. Resistin induces prostaglandin E_2 _and inflammatory cytokines [6]. All of these studies indicate that adipokines can promote cartilage catabolism. However, the mechanism by which these adipokines influence the development of OA is not clearly understood. Recently, elevated levels of extracellular nicotinamide phosphoribosyltransferase (eNAMPT)/visfatin, a newly described adipokine, were reported in plasma and synovial fluid of patients with OA [7,8]. These reports suggest that eNAMPT/visfatin may have a local effect on joint tissue and promote the development of OA.

Nicotinamide phosphoribosyltransferase (NAMPT) is a rate-limiting enzyme in the biosynthetic pathway of nicotinamide adenine dinucleotide [9] and is ubiquitously expressed in many tissues [10]. NAMPT is a 52 kDa protein originally identified as pre-B-cell colony-enhancing factor (PBEF), a cytokine-like protein that stimulated early B-cell formation [11]. NAMPT is a homodimeric protein and is secreted via a secretory pathway independent of the Golgi apparatus and endoplasmic reticulum [12]; NAMPT thus exists in both an intercellular form (iNAMPT) and an extracellular form (eNAMPT) [13]. eNAMPT was renamed recently by Fukuhara and colleagues as visfatin, a visceral fat-derived adipokine that is believed to mimic insulin function [14]. Although binding of NAMPT/PBEF/visfatin to the insulin receptor is debatable, its role in the regulation of insulin secretion in β cells is fairly well established [12]. eNAMPT is thought to be involved in the conversion of nicotinamide into nicotinamide mononucleotide in circulation, which then influences regulation of β-cell function [12]. Interestingly, circulating levels of eNAMPT are elevated in metabolic diseases, including diabetes [15] and obesity [16], and in inflammation [17]. While the function of intracellular NAMPT is well established in the biosynthesis of nicotinamide adenine dinucleotide, the physiological role of extracellular NAMPT is not clear.

Since Fukuhara and colleagues suggested that eNAMPT binds to the insulin receptor and mimics insulin function [14], we sought to examine whether eNAMPT interacts with the insulin-like growth factor-1 (IGF-1) receptor, which has structural similarity with the insulin receptor [18], and mediates IGF-1 function in chondrocytes. IGF-1 is a major growth factor involved in cartilage matrix synthesis and repair. IGF-1 promotes synthesis of collagen type II, proteoglycans (PGs), and other matrix components [19]. Chondrocytes from osteoarthritic cartilage respond poorly to IGF-1 stimulation [20], however, and the underlying mechanism(s) are not clearly understood.

In the present study we examined the effect of eNAMPT in regulating IGF-1 function in chondrocytes. Our data showed that eNAMPT inhibited IGF-1 function by activating the extracellular signal-regulated kinase (ERK)/mitogen activated protein kinase (MAPK) signaling pathway, independent of IGF-1 receptor activation, suggesting a novel mechanism for IGF-1 resistance in OA.

## Materials and methods

### Reagents and antibodies

Collagenase-P was purchased from Roche Applied Science (Indianapolis, IN, USA). Pronase was from Calbiochem (Gibbstown, NJ, USA). DMEM/Ham's F-12 (1:1), antibiotics, fetal bovine serum, and PicoGreen double-stranded DNA assay reagent were from Invitrogen (Carlsbad, CA, USA). IGF-I was from Austral Biologicals (San Ramon, CA, USA). [^35^S]sulfate was from GE Healthcare (Piscataway, NJ, USA). Antibodies and their sources were as follows: insulin receptor substrate (IRS-1) (Ser(P)-312 and total) was from Upstate Biotechnology, Inc. (Lake Placid, NY, USA); Akt (Ser(P)-473 and total), ERK1/2 (Thr(P)-202/Tyr(P)-204 and total) and mitogen-activated protein kinase kinase (MEK) inhibitor (U0126) were from Cell Signaling Technology (Danvers, MA, USA). Recombinant eNAMPT/visfatin/PBEF (endotoxin levels less then < 0.1 EU/μg protein) was from BioVision (Mountain View, CA, USA).

### Chondrocyte isolation and culture conditions

Human ankle cartilage was obtained from tissue donors within 48 hours of death through the National Disease Research Interchange (Philadelphia, PA, USA) and the Gift of Hope Organ and Tissue Donor Network (Elmhurst, IL, USA) in accordance with institutional protocols. Only tissue from donors without a history of known arthritis was used. The tissue was graded on a scale of 0 to 4 for evidence of morphological changes, as previously described [21]. All tissue for this study was either grade 0 or 1. Tissues from a total of 40 donors ranging from 40 to 90 years old were used in the experiments. Cells from at least three independent donors were used in each experiment.

Chondrocytes were isolated under aseptic conditions by sequential enzymatic digestion at 37°C using pronase 2 mg/ml in serum-free DMEM/F-12/antibiotics for 1 hour followed by overnight digestion with collagenase-P at 0.25 mg/ml in DMEM/F-12 (5% fetal bovine serum). Viability of isolated cells was determined using trypan blue and cells were counted using a hemocytometer. Monolayer cultures were established by plating cells in six-well plates at 2 × 10^6 ^cells/ml in DMEM/F-12 medium supplemented with 10% fetal bovine serum. Cells were maintained for approximately 3 to 5 days with feedings every 2 days until they reached 100% confluency prior to experimental use.

### Proteoglycan synthesis assay

The [^35^S]sulfate incorporation assay was performed to measure PG synthesis. Chondrocytes in culture were made serum-free and pretreated with eNAMPT (0 to 5 μg/ml) overnight followed by 24-hour stimulation with IGF-I (50 ng/ml). The medium was then replaced with fresh serum-free medium 1 hour prior to incubation with [^35^S]sulfate for an additional 4 hours. The [^35^S]sulfate incorporation was measured using the Alcian blue precipitation method [15] and normalized to DNA content. DNA was measured using the PicoGreen double-stranded DNA assay according to the manufacturer's protocol.

### ELISA for collagen II

Normal human chondrocytes cultured in serum-free DMEM/Ham's F-12 supplemented with 1% mini ITS plus ascorbate were treated with or without eNAMPT (5 μg/ml) overnight followed by IGF-1 (50 μg/ml) for an additional 24 hours. After incubation, media were removed and cell layers were extracted according to the manufacturer's protocol and analyzed for collagen II levels using an ELISA kit (MD Biosciences Inc., St Paul, MN, USA).

### Quantitative real-time PCR

Total RNA was extracted using TRIzol (Invitrogen) according to the manufacturer's protocol. Total RNA (2 μg) was used to synthesize cDNA using oligo(dT)15 as the reverse primer. Equivalent amounts of cDNA were used for real-time PCR in a 25 μl reaction mixture with 12.5 μl of 2× SYBR Green PCR Mastermix (SA Bioscience, Frederick, MD, USA) and 1 μl specific primer pairs. Reactions were run in triplicate with 40 cycles of amplification on an ABI Prism 7000 real-time PCR machine (Applied Biosystems, Foster City, CA, USA). The sequences of primers used were as follows: TATA box-binding protein, sense (5'-TGCACAGGAGCCAAGAGTGAA-3') and antisense (5'-CACATCACAGCTCCCCACCA-3'); and collagen II, sense (5'-TGCTGCCCAGATGGCTGGAGGA-3') and antisense (5'-TGCCTTGAAATCCTTGAGGCCC-3') [22]. The expression level of collagen II was normalized relative to the expression of TATA box-binding protein measured in parallel samples.

### Chondrocyte stimulation and immunoblotting

Confluent human chondrocyte monolayers were made serum-free overnight before treating with purified recombinant human eNAMPT (0 to 5 μg/ml) overnight followed by stimulation with IGF-1 (50 ng/ml) for 0 to 60 minutes for signaling studies. In some experiments, cells were pretreated with 10 μM MEK inhibitor (U0126) for 30 minutes followed by treatment with eNAMPT or IGF-1. We have previously shown that treatment of cells with MEK inhibitor (U0126) did not affect chondrocyte viability [23]. After incubation, cells were washed with PBS and lysed with lysis buffer that contained 20 mM Tris (pH 7.5), 150 mM NaCl, 1 mM EDTA, 1 mM EGTA, 1% Triton X-100, 2.5 mM tetrapyrophosphate, 1 mM glycerol phosphate, 1 mM Na_3_VO_4_, 1 μl/ml leupeptin, and 1 mM phenylmethylsulfonyl fluoride. Lysates were centrifuged to remove insoluble material, and the soluble protein concentration was determined with BCA reagent (Thermo Scientific, Rockford, IL, USA). Samples containing equal amounts of total protein were separated by SDS-PAGE, transferred to nitrocellulose, and probed for signaling proteins. Immunoreactive bands were detected using the ECL system (GE Healthcare). All immunoblotting experiments were repeated at least three times with similar results.

### Statistical analysis

Data were expressed as the mean ± standard deviation and subjected to analysis of variance using StatView 5.0 software (SAS Institute, Cary, NC, USA). *P *≤ 0.05 was considered significant.

## Results

### Extracellular NAMPT inhibits IGF-1-mediated proteoglycan synthesis

**Figure 1:**
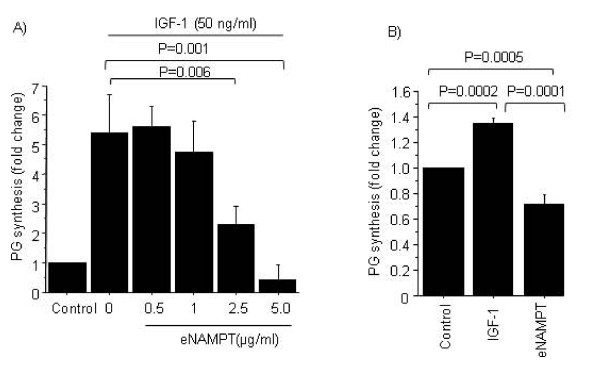
**Extracellular nicotinamide phosphoribosyltransferase inhibits insulin-like growth factor-1-stimulated proteoglycan synthesis. (A) **Normal human chondrocytes were treated with (0 to 5 μg/ml) extracellular nicotinamide phosphoribosyltransferase (eNAMPT)/visfatin overnight, followed by 24-hour stimulation with 50 ng/ml insulin-like growth factor-1 (IGF-1). **(B) **Cells were stimulated with 5 μg/ml eNAMPT or 50 ng/ml IGF-1 overnight. Proteoglycan **(**PG) synthesis was measured using [^35^S]sulfate incorporation and DNA was analyzed with a PicoGreen assay. Results are expressed as the fold change versus unstimulated control. Data presented as mean ± standard deviation of three independent experiments.

We examined the effect of eNAMPT on IGF-1-stimulated PG synthesis. Pretreatment of chondrocytes with eNAMPT overnight, followed by IGF-1 stimulation for an additional 24 hours, inhibited IGF-1-induced PG synthesis. Inhibition by eNAMPT occurred in a dose-dependent manner with maximum inhibition observed at 5 μg/ml (Figure 1A). Interestingly, overnight treatment of chondrocytes with eNAMPT alone inhibited basal PG synthesis (Figure 1B).

### Extracellular NAMPT inhibits the production of collagen type II

**Figure 2:**
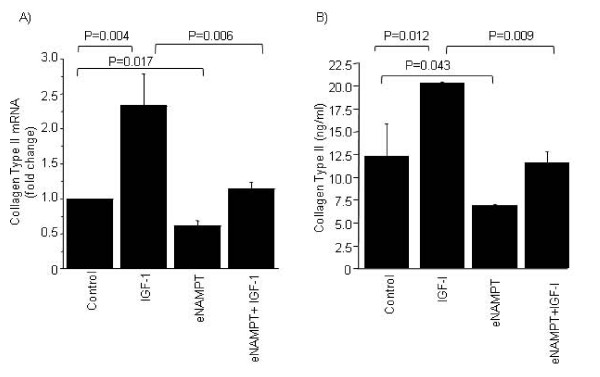
**Effects of extracellular nicotinamide phosphoribosyltransferase on type II collagen expression and synthesis**. Normal chondrocytes were treated with 5 μg/ml extracellular nicotinamide phosphoribosyltransferase (eNAMPT) overnight followed by stimulation with or without 50 ng/ml insulin-like growth factor-1 (IGF-1) for an additional 24 hours. **(A) **Total RNA was isolated, and quantitative real-time PCR was performed to determine mRNA expression of collagen II using the TATA box-binding protein (TBP) as a control. mRNA expression is presented as the fold change relative to control. Data presented as mean ± standard deviation of three independent experiments. **(B) **Cell lysates were collected, and the collagen II protein levels (normalized to DNA) were analyzed by ELISA and presented as the fold change relative to control. Data presented as mean ± standard deviation of three independent experiments.

IGF-1 is known to promote synthesis of collagen type II, a major component of the cartilage matrix. Since we found that eNAMPT inhibits IGF-1-stimulated PG synthesis, we were interested to examine the effect of eNAMPT on collagen type II production. Our data showed that pretreatment of chondrocytes with 5 μg/ml eNAMPT inhibited both basal and IGF-1-stimulated collagen type II expression and synthesis (Figure 2A, B).

### Extracellular NAMPT inhibits IGF-1 signaling in chondrocytes

**Figure 3:**
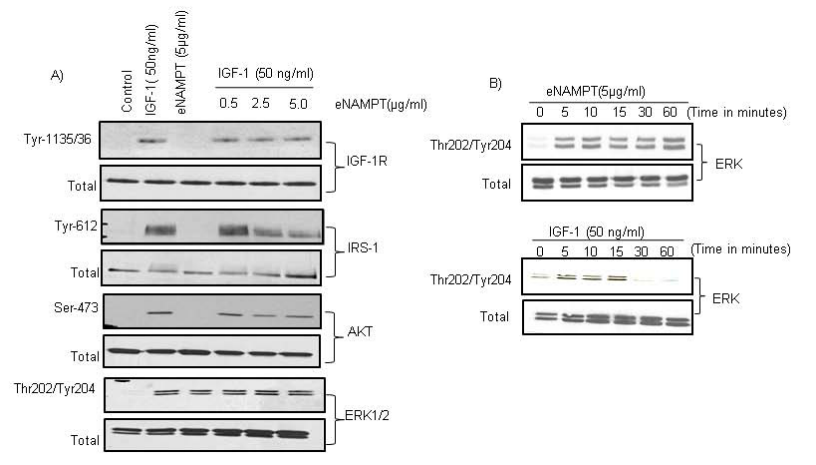
**Effect of extracellular nicotinamide phosphoribosyltransferase on insulin-like growth factor-1 signaling**. **(A) **Chondrocytes were treated with various concentrations (0 to 5.0 μg/ml) of extracellular nicotinamide phosphoribosyltransferase (eNAMPT) overnight and then stimulated with 50 ng/ml insulin-like growth factor-1 (IGF-1) for 10 minutes. Cell lysates were immunoblotted with phosphospecific antibodies to IGF-1 receptor (IGF-1R), insulin receptor substrate-1(IRS-1), AKT, and extracellular signal-regulated kinase (ERK). **(B) **Cells were stimulated with 5 μg/ml eNAMPT or 50 ng/ml IGF-1 for 0 to 60 minutes. After incubation, cell lysates were immunoblotted with total or phosphospecific antibodies to ERK. The immunoblots presented are representative of at least three independent experiments.

Since our data showed that pretreatment of chondrocytes with eNAMPT inhibited IGF-1-mediated PG synthesis and collagen production, we wanted to examine the effect of eNAMPT on IGF-1 signaling. IGF-1-mediated activation of AKT has been shown essential for PG synthesis and collagen type II synthesis [23,24]. Stimulation of normal chondrocytes with IGF-1 resulted in phosphorylation of the IGF-1 receptor (Y-1135/36) and the downstream signaling molecules, including IRS-1 (Y-612; activating site) and AKT (S-473) (Figure 3A, lane 2), whereas eNAMPT alone did not stimulate phosphorylation of IGF-1 receptor, IRS-1 or AKT (Figure 3A, lane 3) but did stimulate a robust and sustained phosphorylation of ERK1/2 (at least for 60 minutes) compared with transient phosphorylation (15 minutes) with IGF-1 (Figure 3B). Pretreatment of chondrocytes with eNAMPT (dosage 0 to 5 μg/ml) followed by IGF-1 stimulation did not change the phosphorylation status of the IGF-1 receptor; however, eNAMPT inhibited the IGF-1-mediated phosphorylation of IRS-1 (Y-612) and downstream AKT (serine-473) (Figure 3A, lanes 4 to 6).

**Figure 4:**
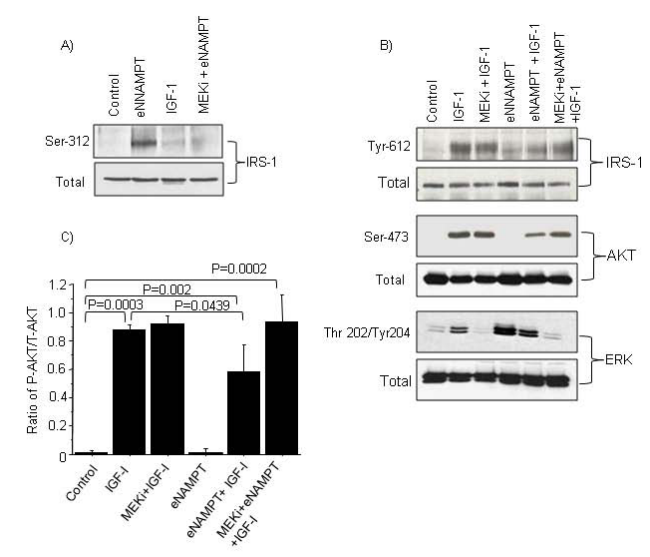
**Extracellular signal-regulated kinase inhibition blocks extracellular nicotinamide phosphoribosyltransferase inhibition of insulin-like growth factor -1 signaling(A), (B) **Cells were pretreated with or without 10 μM mitogen-activated protein kinase kinase inhibitor (MEKi) for 30 minutes followed by treatment with extracellular nicotinamide phosphoribosyltransferase (eNAMPT) overnight, and insulin-like growth factor-1 (IGF-1) for 10 minutes or with IGF-1 alone for 10 minutes. After incubation, cell lysates were immunoblotted with phosphospecific antibodies to insulin receptor substrate-1 (IRS-1), AKT and extracellular signal-regulated kinase (ERK). Blots were stripped and reprobed with nonphosphospecific antibodies. Data are representative of at least three independent experiments. **(C) **The relative AKT (serine-473) phosphorylation level (normalized to total AKT protein) in the treated samples from three independent experiments was determined by densitometry analysis. Data presented as mean ± standard deviation.

In addition, pretreatment of chondrocytes with eNAMPT stimulated increased phosphorylation of IRS-1 at the serine-312 residue (Figure 4A), which is inhibitory to IGF-1 signaling. Pretreatment of chondrocytes with the MEK inhibitor (U1206) inhibited eNAMPT-induced phosphorylation of IRS-1 at the serine-312 residue (Figure 4A) and restored phosphorylation of IRS-1 (Y-612) and AKT (serine-473) equal to the level stimulated by IGF-1 alone (Figure 4B). Since phosphorylation and activation of AKT are important steps in IGF-1-stimulated PG synthesis and collagen expression, we quantified the relative levels of phosphorylated AKT to total AKT from the dataset presented in Figure 4B (see Figure 4C). Our data showed that pretreatment of cells with eNAMPT followed by IGF-1 stimulation decreased AKT phosphorylation by 40%; however, treatment with MEK inhibitor restored IGF-1-induced AKT phosphorylation to 100% (Figure 4C). Taken together, these results suggest that eNAMPT does not directly inhibit the IGF-1 receptor, but activates a separate signaling pathway that results in ERK activation, which then inhibits the IGF-1-mediated activation of IRS-1 and AKT in chondrocytes through serine phosphorylation of IRS-1.

## Discussion

**Figure 5:**
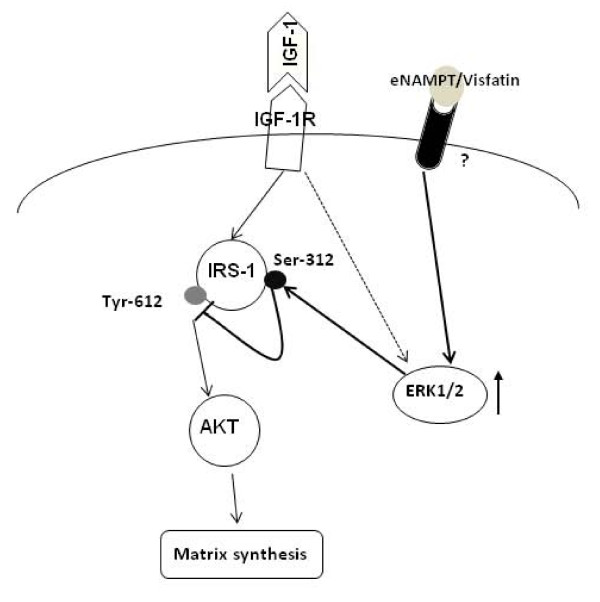
**Model for inhibitory effect of extracellular nicotinamide phosphoribosyltransferase on insulin-like growth factor-1 function in chondrocytes**. In this hypothetical model, sustained activation of extracellular signal-regulated kinases (ERK) by extracellular nicotinamide phosphoribosyltransferase (eNAMPT) promotes increased phosphorylation of insulin receptor substrate-1 (IRS-1) (serine-312), which negatively regulates the downstream insulin-like growth factor-1 (IGF-1) signaling pathway and thereby matrix synthesis. IGF-1R, insulin-like growth factor-1 receptor.

Several earlier studies have indicated a major role for adipokines in cartilage degradation and in the development of OA [25]. However, most studies were focused on the catabolic, not anabolic, pathways of chondrocytes. To the best of our knowledge, this is the first study to examine the effect of an adipokine on IGF-1 function in chondrocytes. We found that eNAMPT/visfatin decreased IGF-1-mediated PG synthesis and collagen production, and this was associated with stimulation of ERK/MAPK and IRS-1 phosphorylation at the serine-312 residue. Increased phosphorylation of IRS-1 at this serine residue has been reported to inhibit IRS-1 tyrosine (serine-612) phosphorylation, resulting in inhibition of downstream phosphorylation of AKT [26,27]. A recent study has shown that chondrocytes produce NAMPT/visfatin, and stimulation of normal chondrocytes with eNAMPT decreased the synthesis of PGs [28]. Taken together, our data suggest that eNAMPT inhibits IGF-1 function and provides a novel mechanism for adipokine-mediated IGF-1 resistance observed in OA chondrocytes (Figure 5).

Binding of IGF-1 to its receptor results in activation of two major signaling pathways, the IRS-1/phosphoinositide-3 kinase/AKT pathway and the ERK/MAPK pathway. Studies have shown that the phosphoinositide-3 kinase/AKT pathway is essential for PG synthesis in chondrocytes, but not the ERK/MAPK signaling pathway, which is inhibitory. Blocking the activation of phosphoinositide-3 kinase or downstream mammalian target of rapamycin inhibits IGF-1-mediated PG synthesis [23]. In our current study, pretreatment of chondrocytes with eNAMPT inhibited IGF-1-induced activation of the IRS-1-AKT signaling pathway while prolonging the activation of the ERK/MAPK pathway. In addition, treatment with eNAMPT also decreased IGF-1-mediated PG synthesis, suggesting that eNAMPT affects the normal function of IGF-1 in cartilage.

We also found that stimulation of chondrocytes with eNAMPT elicited robust and sustained activation of the ERK/MAPK pathway independent of IGF-1 receptor activation. This observation is consistent with an earlier study in human umbilical vein endothelial cells [29], in which eNAMPT activated ERK signaling without activating the insulin receptor. These data suggest that eNAMPT may interact with an unknown receptor and activate a signaling pathway that results in ERK/MAPK activation.

Studies have shown increased ERK activity in chondrocytes isolated from osteoarthritic cartilage [30,31]. Inhibiting ERK/MAPK activation enhanced IGF-1-mediated PG synthesis [24], suggesting that activation of the ERK/MAPK pathway may negatively regulate IGF-I-stimulated PG synthesis. One mechanism by which ERK activity might inhibit IGF-1 signaling is by promoting serine phosphorylation of IRS-1 [32]. Yin and colleagues reported recently that basal phosphorylation of IRS-1 is increased at serine-312 as well as serine-616 in osteoarthritic chondrocytes [24]. In addition, overexpression of constitutively active MEK constructs enhanced the phosphorylation of IRS-1 at the serine residue and inhibited IGF-1-mediated PG synthesis. These studies suggest that increased activation of the ERK/MAPK pathway inhibits IGF-1 signaling. In addition, type II collagen expression was also inhibited by active MEK in previous work, which is consistent with the ability of eNAMPT to decrease collagen expression [24]. Taken together, these studies clearly demonstrate that prolonged activation of ERK/MAPK signaling by eNAMPT is associated with inhibition of IGF-1 function in chondrocytes.

## Conclusions

Our study shows that chondrocytes respond to eNAMPT stimulation with sustained activation of the ERK/MAPK pathway, independent of IGF-1 receptor activation. Increased ERK activity results in decreased IGF-1 function in chondrocytes, and thus could contribute to IGF-1 resistance in osteoarthritic tissues.

## Abbreviations

DMEM: Dulbecco's modified Eagle's medium; ELISA: enzyme-linked immunosorbent assay; eNAMPT: extracellular nicotinamide phosphoribosyltransferase; ERK: extracellular signal-regulated kinase; IGF-1: insulin-like growth factor-1; IL: interleukin; IRS-1: insulin receptor substrate-1; MAPK: mitogen-activated protein kinase; MEK: mitogen-activated protein kinase kinase; NAMPT: nicotinamide phosphoribosyltransferase; OA: osteoarthritis; PBEF: pre-B-cell colony-enhancing factor; PBS: phosphate-buffered saline; PCR: polymerase chain reaction; PG: proteoglycan.

## Competing interests

The authors declare that they have no competing interests.

## Authors' contributions

RRY designed the study, acquired the data, analyzed and interpreted the data, and prepared the manuscript. RFL designed the study, interpreted the data, and helped with the manuscript preparation. Both of the authors have final approval of the manuscript.
